# Supporting Decision Making in Intensive Care: Ethical Principles for Managing Access to Care During the COVID-19 Pandemic

**DOI:** 10.3389/fmed.2021.787805

**Published:** 2021-12-03

**Authors:** Stefano D'Errico, Martina Padovano, Matteo Scopetti, Federico Manetti, Martina Zanon, Alessandro Santurro, Paola Frati, Vittorio Fineschi

**Affiliations:** ^1^Department of Medicine, Surgery, and Health, University of Trieste, Trieste, Italy; ^2^Department of Anatomical, Histological, Forensic and Orthopaedic Sciences, Sapienza University of Rome, Rome, Italy; ^3^Department of Medicine, Surgery and Dentistry, University of Salerno, Salerno, Italy; ^4^Istituto di Ricovero e Cura a Carattere Scientifico (IRCCS), Neuromed, Pozzilli, Italy

**Keywords:** COVID-19 pandemic, global health emergency, access to care, intensive care, ethics

## Abstract

The pandemic from COVID-19 causes a health threat for many countries and requires an internationally coordinated response due to the high spread of the infection. The current local and international situation gives rise to logistical and ethical considerations regarding the imbalance between needs for assistance and availability of health resources in the continuation of the emergency. A shortage condition will require healthcare professionals to choose between patients who will have access to respiratory support and those who will have to continue without. The sharing of criteria for the introduction of patients to the different therapeutic paths is fundamental to prevent the onset of ethical issues. The present paper analyzes the critical issues related to the scarcity of healthcare resources and the limitation of access to intensive care with the aim of proposing ethically sustainable principles for the management of the current pandemic situation.

## Introduction

The declaration of pandemic from COVID-19 released by the World Health Organization (WHO) on March 11, 2020 sanctioned the beginning of a public health emergency of international concern (PHEIC). This state of emergency causes a health threat for many countries and requires an internationally coordinated response due to the high spread of the infection (1).

The increase in contagions has forced different governments to adopt drastic containment measures. In the Italian context, the emergency response implied the total closure of all non-essential activities and the prohibition on all people to move except for proven work needs, absolute urgency or for health reasons.

The current local and international situation gives rise to logistical and ethical considerations regarding the imbalance between needs for assistance and availability of health resources in the continuation of the emergency. In fact, the outbreak of the novel Coronavirus (nCoV) engaged human and material resources far beyond the tolerance limits of health systems, producing conditions of extraordinary lack of care (2).

The management of ethical disputes in the emergency period requires advance planning that provides specific guidelines to hospital management and healthcare professionals. Among the challenges to be addressed, limiting access to intensive care represents the main and most discouraging issue. The severity and duration of respiratory problems due to COVID-19 is able to saturate the intensive care system in a very short time (3). A shortage condition requires healthcare professionals to choose between patients who will have access to respiratory support and those who will have to continue the therapeutic course without.

The sharing of criteria for the introduction of patients to the different therapeutic paths is fundamental precisely to prevent the onset of ethical issues when the time for evaluating alternatives will necessarily be scarce (4).

The present paper analyzes the critical issues related to the scarcity of healthcare resources and the limitation of access to intensive care with the aim of proposing ethically sustainable principles for the management of the current pandemic situation.

## Issues in Access to Intensive Care

The current pandemic scenario has led to a serious shortage of respiratory support machines requiring a rapid “arms race” for crisis management. According to previous estimates, a pandemic may require tripling the availability of mechanical ventilators (5).

System overload is made it impossible to provide respiratory support to patients with respiratory failure who need mechanical ventilation to survive. The deficiency status raised unprecedented allocation dilemmas that imposed the subordination of any decision to public health goals (6). As well as beds and drug therapies, respiratory support should be considered a resource to be rationed and assigned based on criteria established in the interest of public health rather than decisions of individual doctors and patients (7). The formulation of guidelines for the allocation of medical resources in a condition of scarcity requires multiple interventions characterized by different levels of specificity. At a more general level, national health policies must express a social agreement on the need to link the decisions of individual doctors and patients to public health needs during the emergency phase. At a specific level, regarding decisions on clinical care, hospitals, and healthcare professionals must share criteria for managing access to intensive care when demand significantly exceeds supply. Finally, the professionals in the front line must have clear guidelines for the implementation of the triage process and the decision between the different care paths in specific cases (8). In principle, for the optimization of care and the reduction of deaths during an emergency, priority should be given to patients who need mechanical ventilation but who are very likely to survive after a few days of respiratory support.

Under normal conditions, the lack of resources for intensive care constitutes a sporadic event that can be resolved through an assignment based on the “first come, first served” principle. Sporadic shortages generally lead to the development of short-term measures to increase the availability of intensive care. Among other things, it is important to divert patients to other hospitals, cancel elective surgery, use post-operative rooms as temporary Intensive Care Unit (ICU) beds. Furthermore, it is possible to accelerate the transfer to the ward of patients weaning from intensive care to maximize the availability of ventilators. It is absolutely clear how such measures cannot be feasible during the present health emergency precisely because of the severity and duration of the current coronavirus disease.

The emergency context requires instead to review the general principles for the allocation of scarce resources with a view to maximizing health outcomes, giving priority to patients who can be treated more efficiently (9). Although in general the ethical line to follow is to help the neediest patients by maximizing the number of lives saved, in a restriction period there may be a contradiction with the principles of allocation (10). Therefore, it is essential to establish an agreement on the principles to be applied for the management of resources in the emergency phase (11).

The first ethical issue concerns the possibility that the goal of maximizing the number of lives saved could take over the patient's autonomy. Public health officials, clinical experts, and political representatives should agree on criteria for establishing the care priorities that individual healthcare professionals and patients should follow.

Secondly, it is not disputed that patients with a high probability of survival after a few days of intensive care should receive the highest priority. However, the characterization of such a group of patients is extremely difficult since the evidence is currently scarce and incomplete (12); in fact, for example, there is no data to predict the duration of intensive care (13). The scarcity of evidence requires reaching a consensus based on the discussion of available data and expert opinion.

The third issue is related to equity and perception of equality during a public health emergency. The population is more inclined to subordinate personal interest to the common good if the constant application of the same criteria is evident. Specifically, people are less likely to accept mandatory emergency measures and to sacrifice for the community if apparently some are receiving special consideration or favoritism (14).

Finally, the fourth problem concerns the obligation to ensure transparency during the emergency phase. The priorities and policies of triage should be accessible in order to make the methods for allocating resources known to the population and prepare the community for any individual discussions on access to care (15).

Although there is a broad consensus on the use of triage to minimize loss of life during a pandemic, hospitals, and healthcare professionals are forced to face heterogeneous situations and make difficult decisions in specific cases (16).

The complexity of the problem is increased by the need to re-evaluate patients who have already had access to intensive care. In fact, patients receiving respiratory support may have a worse prognosis than new patients with respiratory failure. The continuation of intensive care in patients with poor prognosis and low expectations of maintaining the state of health determines a limitation of access for patients who instead, despite being able to benefit from respiratory support, are directed toward other care paths.

Therefore, the non-inclusion of patients already admitted to intensive treatment in the triage process can lead to a decrease in the total number of lives saved. On the other hand, discontinuing intensive care for patients with poor prospects for improvement would violate the ethical rules that the physician should be loyal to patients and act in their best interest. Although the choice to stop treatment is emotionally difficult for healthcare professionals and the patient, logically there is no difference between the interruption and the initial exclusion if in both cases the justification complies with the emergency rules and has been discussed between the subjects involved.

With a view to profitable crisis management, it may be useful to separate the roles of triage and care to allow doctors to keep the patient's interests a priority. An out-of-care physician in the intensive care unit can be appointed to make triage decisions so that the doctors involved in care are not obliged to decide to maintain or stop mechanical ventilation. Such an approach creates a situation in which the triage doctor can make decisions based on the overall results for the population, while the attending physician is free to serve the patient's best interest.

If the shortage of means for intensive care persists after the application of the probability of survival and short-term need for mechanical ventilation criteria, several other criteria may be considered for the assignment of respiratory support. These criteria could include life expectancy and likely quality of life after treatment. However, the use of such criteria should be limited or even avoided in emergency conditions due to the physician's evaluative subjectivity, possible disagreements with the patient and concerns about injustice.

Unfortunately, patients with respiratory failure who do not have access to ventilatory support can experience death. Therefore, such patients should be candidates for respectful and compassionate palliative care pathways, including at home (17). Death from respiratory failure can be extremely distressing because of the feelings of drowning and air hunger to which it is related. The administration of sedatives and analgesics is to be considered ethically and clinically appropriate in such situations, even at doses capable of causing loss of consciousness if lower doses fail to alleviate the symptoms. Although palliative sedation has a strong ethical justification, not all healthcare professionals are trained in palliative sedation and the reduction of hospital supplies can cause a shortage of the drugs needed to alleviate the symptoms.

## Position Statement

**Figure 1:**
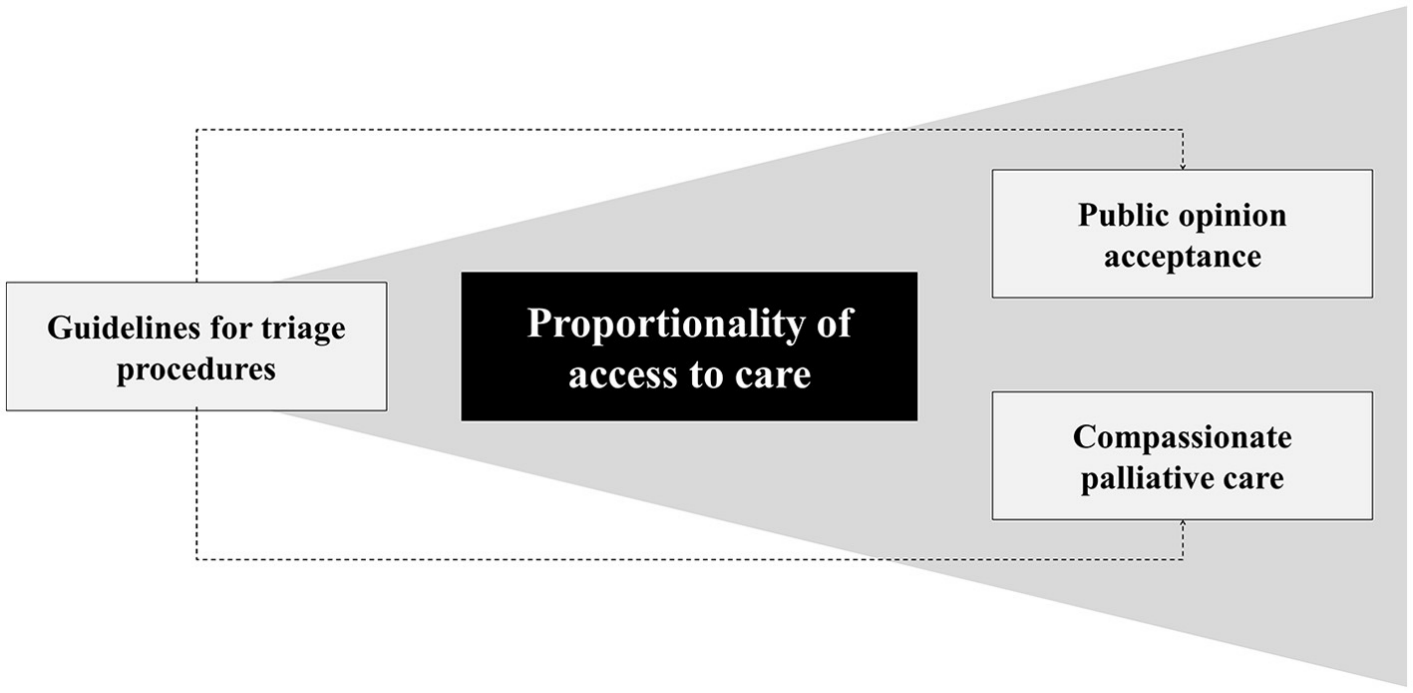
Position statement on proportionality of access to care during pandemic.

The response of health systems to the pandemic emergency imposes medical, scientific, moral, and ethical considerations on the political and health authorities involved (Figure 1). The review of existing triage procedures to meet the overwhelming demand for intensive care requires the responsible application of the principles of equity, justice, usefulness, efficiency, transparency, and participation.

Preliminarily it is essential to establish that the scope of the guidelines for the selection of access to intensive care must be universally limited to contexts in which there is an effective scarcity of health resources. In fact, the aprioristic application of the triage procedures would be in clear contradiction with the previously mentioned principles.

Governments and healthcare systems must provide hospitals and healthcare professionals with explicit criteria for assessing patients with respiratory failure under conditions of limited access to intensive care. Similarly, it is essential to prepare guidelines for the practical management of critical issues during the implementation of triage procedures.

For a correct allocation of resources, it is essential to aim at maximizing therapeutic successes and safeguarding public health by prioritizing patients who can be treated in a more profitable and efficient way. In this perspective, it is important to plan health care according to the integration between home and hospital care through the formulation of shared operational protocols; in this way, it would be possible to identify patients manageable at home avoiding the excess of requests for hospitalization. Such an objective presupposes the implementation of territorial systems through the recruitment of health professionals and specific training.

Triage procedures should be conducted by professionals not directly involved in patient care. Based on the criticality of the conditions at the local level, the triage procedure should be extended by re-evaluating the subjects already admitted to intensive care.

In view of the objective difficulties weighing on the prognostic evaluation of patients, the recommendations should be promptly updated based on the evidence on COVID-19.

Nonetheless, the regulatory obligation to inform patients on decision-making criteria emerges to foster trust in care relationships. Particular attention must be paid to fragile individuals who may be at risk of family and social isolation at the time of need for assistance. Sharing the established criteria with public opinion is fundamental in order to promote acceptance of the sacrifice for the benefit of public health and limit discussions during the management of concrete cases.

Of course, given the impossibility of guaranteeing access to intensive care for all patients, it is necessary to plan paths for the provision of respectful and compassionate palliative care. The administration of analgesic and sedative drugs should however be carried out maintaining the objective of alleviating suffering and avoiding active euthanasia practices.

## Discussion

The outbreak and rapid evolution of the COVID-19 pandemic forced national health systems to redefine the priorities for access to care due to the increased need for assistance and the scarcity of resources. The present discussion has outlined the general principles that should be considered in the management of the current pandemic emergency for the protection of public health.

The main question in a pandemic situation concerns compatibility between the restrictions imposed by the need to allocate health resources and the assistance obligations of health systems. Specifically, it is necessary to establish whether in a context of rationing it is necessary to review the ethical principles underlying medical care. Obviously, ethical foundations of care must change in consideration of the dramatic increase in health care loads imposed by the pandemic (18).

Certainly, the ordinary “first come, first served” criterion must deal with the critical issues related to the scarcity of resources making it necessary to adopt practical choices (19). Therefore, any evaluation should be carried out considering the probabilities of benefit, the possibilities of improving the quality of life, the expected effectiveness of the measures taken, the critical condition of the patient, as well as—secondly—the resources required for the success of the treatment (20).

The extraordinary nature of the pandemic scenario, especially in the acute phases of the different waves, cannot lead to the overcoming of fundamental ethical values. The COVID-19 health emergency highlighted the importance of promoting macro-allocation policies capable of guaranteeing the protection of the individual even in exceptional conditions. In other words, the implementation of health policies aimed at investments in preparation is fundamental so that, in the emergency state, one should not be forced to choose which individuals to treat. What happened during the pandemic must lead to a reflection on the protection of the individual and on the need for maximum inclusion in care pathways so that the rationing or scarcity of available resources should not lead to the identification of criteria for selecting the value of human life. Ultimately, it is crucial to avoid “loosening of the mesh,” even if only temporary, of the protection network of the fragile individual to avoid the risk of marginalization, discrimination, and disproportionality in access to care, even outside the emergency. The goal of health systems must be to ensure the greatest number of lives saved, guarantee life expectancy and aim to improve the quality of life. In this perspective, it is necessary to ask whether the health professional can independently be a resource allocator and whether he can alone make choices that penalize the most vulnerable people (21).

Considering that the right to health is universally thought fundamental, inalienable, and essential for the dignity of life, the need to support health professionals in the emergency context is evident, making sure that the individual is not forced to decide for himself which patient to sacrifice.

## Data Availability Statement

The original contributions presented in the study are included in the article/supplementary material, further inquiries can be directed to the corresponding author/s.

## Author Contributions

All authors contributed equally to manuscript drafting, critical discussion, and approved the final version.

## Conflict of Interest

The authors declare that the research was conducted in the absence of any commercial or financial relationships that could be construed as a potential conflict of interest.

## Publisher's Note

All claims expressed in this article are solely those of the authors and do not necessarily represent those of their affiliated organizations, or those of the publisher, the editors and the reviewers. Any product that may be evaluated in this article, or claim that may be made by its manufacturer, is not guaranteed or endorsed by the publisher.
